# Frequent KIT mutations in skin lesions of patients with BRAF wild-type Langerhans cell histiocytosis

**DOI:** 10.1007/s00428-020-02820-w

**Published:** 2020-05-05

**Authors:** Béla Tóth, Norbert Kiss, Judit Hársing, Sarolta Kárpáti, Judit Csomor, Csaba Bödör, József Tímár, Erzsébet Rásó

**Affiliations:** 1 Department of Dermatology, Venereology and Dermatooncology, Semmelweis University, 41 Mária utca, Budapest, H-1085 Hungary; 2 1st Department of Pathology and Experimental Cancer Research, Semmelweis University, Budapest, Hungary; 3 2nd Department of Pathology, Semmelweis University, Budapest, Hungary

**Keywords:** Langerhans cell histiocytosis, BRAF, MAP2K1, NRAS, KIT

## Abstract

Langerhans cell histiocytosis (LCH) is characterized by mutations of the RAS-RAF-MAPK signaling pathway. We analyzed MAP2K1, NRAS and KIT mutation incidence in skin lesions of BRAF wild-type (wt) LCH patients. We evaluated the occurrence of MAP2K1, NRAS and KIT mutations in seven LCH and one indeterminate cell histiocytosis (ICH) patients. MAP2K1 mutation frequency was found to be 3/7 (42.9%) in LCH and also found in ICH. Similarly, the KIT mutation frequency was found to be equally prevalent (4/7, 57.1%) in LCH and also occurred in ICH. Involvement of KIT exons in LCH-ICH indicated that exon 9/11/18 were equally prevalent followed by exon 13. This exploratory analysis on BRAF-wt LCH revealed a KIT mutation rate comparable to MAP2K1. Although the detected KIT mutations are different from activating mutations found in other KIT-dependent neoplasms, our data suggest that KIT-inhibitors might have a role in treating BRAF-wt LCH patients.

## Introduction

The revised classification scheme of the Histiocyte Society classified histiocytic disorders into five groups. Group L (Langerhans) includes Langerhans cell histiocytosis (LCH), indeterminate cell histiocytosis (ICH), Erdheim-Chester disease (ECD) and mixed LCH/ECD [1]. The incidence of LCH, which is the most common histiocytic disease, is estimated to be between 4.6 and 9 cases per million children, while it is 1–2 cases per million adults [2]. The most frequent mutational driver in L type histocytoses is BRAF, which rarely affects ARAF. This is followed by frequent mutations of MAP2K1 (rarely MAP3K1). Mutations rarely affect RAS itself (N- or H-) or the AKT-mTOR signaling pathway [1, 2].

Different therapeutic strategies are used for the treatment of histiocytosis, usually resulting in an excellent control of the localized disease, but the multifocal or systemic disease can be refractory to contemporary therapies [3]. BRAF inhibitors have been successfully tested in LCH [4]. Since the protein expression of c-KIT was accidentally demonstrated in LCH, this prompted clinical studies. While KIT-inhibitor imatinib mesylate was successfully used in certain cases of LCH [5], KIT mutations have not been demonstrated in the skin lesions. Accordingly, our aim was to analyze KIT mutations in skin lesions of patients with LCH carrying wild-type (wt) BRAF.

## Methods

### Patients and sample analysis

Formalin-fixed, paraffin-embedded skin samples of six adult and two pediatric patients with histiocytoses belonging to group L (LCH: seven patients, ICH: one patient) were evaluated. All samples were collected from the histologic archive of the Department of Dermatology, Venereology and Dematooncology, Semmelweis University, Hungary. All patients were diagnosed and treated at the department between 2003 and 2015. The following inclusion criteria were applied: (1) tissue specimens included > 10% tumor infiltration and (2) wt BRAF with Sanger sequencing. The patients’ characteristics are shown in Table 1. The study was conducted in accordance with the Declaration of Helsinki and was approved by the Science and Research Ethics Committee of Semmelweis University.

**Table 1:** Patient characteristics

Patient number	Age (years)	Gender	Diagnosis	Cutaneous lesions			Systemic involvement	Treatment	Treatment outcome
				Type of the lesions	Location of the lesions	Single or multiple lesions			
1	45	Male	LCH (L gr)	Papules	Scalp, face, genital area, trunk	Multiple	None	Irradiation	Improved
2	36	Male	LCH (L gr)	Papules, plaques, erosions	Scalp, intertriginous areas	Multiple	Endocrine system (diabetes insipidus–symptoms, laboratory)	Thalidomide	Improved
3	22	Female	LCH (L gr)	Papules	Scalp	Multiple	Lung	Lung transplant, thalidomide	Improved
4	71	Female	LCH (L gr)	Papules	Scalp	Multiple	None	Topical steroid	Improved
5	46	Male	LCH (L gr)	Pruritus, papules, erosions	Scalp, face, trunk, intertriginous areas, genital area, legs	Multiple	Endocrine system (thyroid gland-histology)	Thalidomide	Improved
6	1	Male	LCH (L gr)	Papules, pustules	Face, extremities, fingers	Multiple	Lymph nodes, spleen (histology)	Cytostatic treatment	Recurrent
7	79	Female	LCH (L gr)	Pruritus, papules	Trunk	Multiple	None	Topical steroid	Improved
8	15	Male	ICH (L gr)	Papules	Trunk, extremities	Multiple	None	Thalidomide	Improved

The diagnosis was based on the histologic evaluation of hematoxylin-eosin-stained sections, S100b, CD1a, CD68, and langerin (CD207) immunohistochemistry, and ultrastructural microscopy. All samples were re-reviewed by two independent pathologists according to the revised classification of the Histiocyte Society [1].

### Detecting BRAF, NRAS, KIT, and MAP2K1 mutations

Following deparaffinization, macrodissections were performed on all tissue sections to select tumor cell-rich regions. DNA samples were isolated using High Pure PCR Template Preparation Kit (Roche Diagnostics GmbH, Germany) according to the protocol (Version 20). Exon 3 of the NRAS, exon 15 of the BRAF, exons 2 and 3 of the MAPK2K1 gene and exons 9, 11, 13, 17, and 18 of the KIT gene were amplified by PCR using the following oligonucleotide primer pairs (designed with Array Designer-BiosoftInternational): NRASe3S: AAACAAGTGGTTATAGATGGTGAAAC; NRASex3A: GTAGAGGTTAATATCCGCAAATGAC; BRAFe15S: TTCCTTTACTTACTACACCTCAGA; BRAFe15A: TGGAAAAATAGCCTCAATTC; MAP2K1e2S: GTGACAGTATTGACTTGTGCTC; MAP2K1e2A: AGTCTTCCTTCTACCCTGGTC; MAP2K1e3S: TCATCCCTTCCTCCCTCTTTC; MAPK2K1e3Ab: CTCGCCATCGCTGTAGAAC; KIT9S: AAGTATGCCACATCCCAAGTG; KIT9A: GGTAGACAGAGCCTAAACATCC; KIT11S: CAGAGTGCTCTAATGACTGAGAC; KIT11A: AAGCCACTGGAGTTCCTTAAAG; KIT13S: CTTGACATCAGTTTGCCAGTTG; KIT13A: TCCAAGCAGTTTATAATCTAGCATTG; KIT17BS: AAAAGTTAGTTTTCACTCTTTACAAG; KIT17BA: CTTAATTTGACTGCTAAAATGTGTG; KIT18S: TCAGCAACAGCAGCATCTATAAG; KIT18A: CAAGGAAGCAGGACACCAATG. The PCR reaction mixture contained 12.5 μl AmpliTaqGold® 360 Master Mix (Thermo Fisher Scientific, MA, USA), 2.5–2.5 μl of the appropriate primer pair, 2 μl of the cDNA and 5.5 μl DEPC treated water for the final volume of 25 μl. The cycling conditions for all primers were 97 °C for 10 min once, then 95 °C for 1 min, 55 °C for 1 min, 72 °C for 2 min for 35 cycles, 72 °C for 10 min.

PCR products were separated on 2% agarose gel and captured with MULTI GENIUS Bio Imaging System (Syngene, MD, USA) after ethidium bromide staining. The bands were excised, and DNA was purified using the EZ-10 Spin Column DNA Gel Extraction Kit (Bio Basic Inc. Canada).

### Sanger sequencing

The purified PCR fragments of PCR products were analyzed by direct sequencing in both sense and antisense directions. The sequencing reaction was done with BigDye® Terminator v1.1 Cycle Sequencing Kit (Applied Biosystems™-Thermo Fisher Scientific) according to the manufacturer’s protocol (same primers as the ones used for PCR amplification reactions). Before analysis, the purification of the sequencing reaction products was done with BigDye® XTerminatorTM Purification Kit (Applied Biosystems™–Thermo Fisher Scientific). PCR products were analyzed by a four-capillary automated sequencer (3130 Genetic Analyzer, Applied Biosystems™).

## Results

Three out of seven BRAF-wt LCH patients in this cohort were found to have MAP2K1 mutations (3/7, 42.9%). It is of note that the ICH tumor also carried a mutant MAP2K1. Furthermore, in two out of these four MAP2K1 mutant samples, SNPs of MAP2K1 were also detected (Table 2).

**Table 2:** Mutational status of L histiocytoses

Patient number	Diagnosis	BRAF status	KIT status					MAP2K1 status		NRAS status
			Exon 9	Exon 11	Exon 13	Exon 17	Exon 18	Exon 2	Exon 3	Exon 3
1	LCH	wt	*p.V473M*	**p.K558K**	wt	wt	*p.P832S*	wt	wt	wt
2	LCH	wt	wt	wt	wt	wt	wt	wt	wt	wt
3	LCH	wt	wt	wt	**p.A636A**	wt	*p.V833M*	**p.L37L**, p.K64*	wt	wt
4	LCH	wt	p.Q459*	wt	wt	wt	wt	wt	wt	wt
5	LCH	wt	wt	*p.P585L*	*p.H650Y*	wt	wt	**p.L37L**	wt	wt
6	LCH	wt	wt	wt	wt	wt	**p.S854S**	wt	*p.E102_I103del*	wt
7	LCH	wt	wt	**p.Y571Y**	**p.H650H**	wt	wt	p.Q34*, p.R47*, **p.V58V**	wt	wt
8	ICH	wt	wt	*p.V569I*	wt	wt	wt	*p.G79D, p.K157R*	wt	wt

*ICH* indeterminate cell histiocytosis, *LCH* Langerhans cell histiocytosis

Clinically relevant mutations are shown in italics; SNPs are in bold

KIT mutations were detected in four BRAF-wt LCH samples out of seven (57.1%) and the ICH sample was also positive for KIT mutation. We found seven KIT mutations in the five mutant histiocytosis cases. Mutations were mapped to exons 9 (2/7, 28.6%), 11 (2/7, 28.6%), 13 (1/7, 14.3%), and 18 (2/7, 28.3%) (Table 2). Except for one mutation, which was a nonsense mutation, all mutations were identified as missense mutations. In two LCH patients (patient Nos. 1 and 5), two KIT exons were involved in parallel: exons 9, 18 and exons 11, 13, respectively. SNP frequency of KIT was also similarly frequent among the BRAF-wt LCH (4/7, 57.1%). Interestingly, two out of four of the KIT-SNPs occurred together with KIT mutations in LCH. It is of note that one KIT-SNP, exon 11 K558K found in LCH is considered silent driver (COSM1243). Furthermore, all samples were found to be wt for NRAS (Table 2).

The KIT mutant LCH patient with endocrine involvement (patient No. 5) responded to thalidomide treatment with partial regression. KIT mutant cutaneous LCH cases (patient Nos. 1 and 4) responded well to topical steroids and soft-X-ray irradiation. The LCH patient carrying both MAP2K1 and KIT mutations with lung involvement (patient No. 3) showed significant improvement upon lung transplantation and thalidomide treatment.

## Discussion

Our results suggest that LCH may belong to the group of KIT-mutant tumors, GIST [6], mastocytosis [7], AML [8], and melanoma [9]. Here, we show novel KIT mutations in 57.1% of BRAF-wt LCHs. Mutations of exons 9, 11, 13, and 18 encoding the extracellular, juxtamembrane and kinase domains were demonstrated (Table 3). Most of the detected KIT mutations are non-classic except exon 9 extracellular domain (ECD) deletion. A similar ECD deletion was reported in AML involving exon 8, it was considered to be an activating one with imatinib sensitivity [10]. Three other point mutations (exon 9 p.V473M, exon 11 p.V569I, exon 18 p.V833M) are considered to be pathogenic by COSMIC database and FATHMM analysis [14]. A further three point mutations (exon 11 p.P585, exon 13 p.H650, exon 18 p.P832) have been found to be loss of function mutations in piebaldism, a KIT-mutant pigmentation/melanocyte disorder [11–13]. It might be important that these point mutations of KIT in histiocytosis occurred together in two cases (patient Nos. 1 and 5) (Table 2). The MAP2K1 mutation frequency in BRAF-wt LCHs was 42.9%, confirming the data of previous reports [1]. Moreover, KIT mutations were not mutually exclusive with MAP2K1 as in two out of five KIT-mutant cases, MAKP2K1 mutations were concomitant. It is of note that in these cases, the concomitant KIT mutations are pathogenic/activating (patient Nos. 3 and 8) [14]. Since we have detected KIT mutation in the histiocytic infiltration of a patient with ICH together with MAP2K1 mutation, this suggests a genetic similarity to LCH.

**Table 3:** Mutational spectrum of KIT in histiocytosis

Exon	Domain	SNV	Chromosome	Position°	Variant	FATHMM		Reference	Other driver
						Prediction	Score		
9	ECD	p.Q459*	4	54,725,888	C/T	Benign	0.14	ref. [10]	
		p.V473M	4	54,725,927	G/A	Pathogenic	0,84	COSM1736818	KIT exon18 p.P832S LOF
11	JM	p.V569I	4	54,727,473	G/A	Pathogenic	0.93	COSM144154	MAP2K1 exon2 mutation
		p.P585L	4	54,727,522	C/T	Pathogenic	0.94	ref. [11]	KIT exon13 p.650Y LOF
13	TK1	p.H650Y	4	54,728,079	C/T	Pathogenic	0.94	ref. [12]	KIT exon11 p.P585L LOF
18	TK2	p.P832S	4	54,736,507	C/T	Pathogenic	0.95	ref. [13]	KIT exon9 p.V473M
		p.V833M	4	54,736,510	G/A	Pathogenic	0.96	(ref. [14])	MAP2K1 exon2 mutation

°GRCh38/hg3

*FATHMM-XF* enhanced accuracy in predicting the functional consequences of non-coding and coding single nucleotide variants (SNVs), *COSM* COSMIC database, *ECD* extracellular domain, *JM* juxtamembrane, *LOF* loss of function, *SNV* single-nucleotide variant, *TK* tyrosine kinase

http://fathmm.biocompute.org.uk/fathmm-xf/

Imatinib has been successfully used in certain cases of LCH [5]. Our data indicate that BRAF-wt LCH patients frequently carry somatic KIT mutations which are different from the classic hotspot mutations of imatinib-sensitive KIT-mutant tumors [15]. This might suggest a therapeutic potential of KIT inhibitors in the case of pathogenic/activating KIT mutations exclusively. Since these KIT mutant LCHs frequently contain concomitant MAP2K1 mutations as well, dual inhibition of KIT and MEK could theoretically be a novel therapeutic option in the future. However, further studies are needed to define the functional and therapeutic consequences of the found mutations of LCHs.
